# Group affiliation in self‐management: support or threat to identity?

**DOI:** 10.1111/hex.12448

**Published:** 2016-02-12

**Authors:** Dagmara Bossy, Ingrid Ruud Knutsen, Anne Rogers, Christina Foss

**Affiliations:** ^1^ Norwegian National Advisory Unit on Learning and Mastery in HealthAker SykehusUniversitetssykehus HFOsloNorway; ^2^ Department of Nursing ScienceFaculty of MedicineUniversity of OsloOsloNorway; ^3^ NIHR CLAHRC WessexFaculty of Health SciencesUniversity of SouthamptonHampshireUK

**Keywords:** group‐based self‐management support, institutional logic, self‐management, type 2 diabetes

## Abstract

**Background:**

Self‐management is considered important in chronic illness, and contemporary health policy recommends participation in support groups for individuals with chronic conditions. Although withdrawal from or non‐participation in support groups is an important problem, there is limited knowledge about individuals' own motivation for participation in or withdrawal from self‐management support groups.

**Objectives:**

To investigate how individuals with type 2 diabetes perceive participation in group‐based self‐management support.

**Design:**

This is a qualitative focus group study using a semi‐structured interview guide.

**Setting and participants:**

Sixteen participants diagnosed with type 2 diabetes were included in the study. Individuals with and without group affiliations were mixed in three focus groups to trigger discussions. In the analysis, reoccurring themes of engagement and discussions between participants were focused within a theoretical frame of institutional logic. The focus groups are seen as social spaces where participants construct identity.

**Results:**

Both participation and non‐participation in group‐based self‐management support are associated with dealing with the stigma of having type 2 diabetes. Negotiations contribute to constructing an illness dignity as a response to the logic of moral responsibility for the disease.

**Discussion and conclusion:**

Contemporary policy contributes to societal understandings of individuals with type 2 diabetes as morally inadequate. Our study shows that group‐based self‐management support may counteract blame and contribute in negotiations of identity for individuals with type 2 diabetes. This mechanism makes participation in groups beneficial for some but stigma inducing for others.

## Introduction

This study focuses on how people with type 2 diabetes perceive participation in group‐based self‐management support. The rising prevalence of chronic illness has required Western societies to adapt their policies to meet a growing demand for long‐term condition management. The idea of individuals self‐managing their own condition has assumed a growing salience in contemporary health policy, and consequently, supporting self‐management is considered to be a central component of care.^1^, ^2^, ^3^ In line with a wider individualization trend in society, patients as self‐managers represent a shift from collective expectations to those of the individual, promoting the logic of moral responsibility and implying a strong normative ethos focused on health‐related behaviours.^4^, ^5^, ^6^ Type 2 diabetes is an increasingly prevalent condition that is largely associated with self‐management requirements, including monitoring, diet and exercise.^7^ The demands of the self‐management of type 2 diabetes are described as challenging,^8^ and inadequate health behaviours may induce feelings of shame and guilt in this patient group.^9^

To support individuals with chronic conditions, group‐based self‐management support has been initiated and is highly regarded in Western health policy as a cost‐effective way to enhance health.^10^ A broad definition of self‐management support involves care and support from friends and wider community ties,^11^ including group‐based activities, such as participating in an association, voluntary self‐management support groups, or shared lay and professional education groups. The focus of our study is to asses how group‐based self‐management support is perceived by individuals with type 2 diabetes in Norway.

The literature highlights that the sharing of patient experiences in groups contributes to the construction of a highly valued collective illness identity that challenges traditional medical knowledge.^12^ Shared lay and professional group education is described as means of achieving success in correcting erroneous health understandings and teaching specific clinical disease management skills.^13^ Groups of peers provide effective emotional support through building trust, fostering friendship and providing reassurance.^14^, ^15^

Group‐based support involving professionals and lay representatives has also been the subject of criticism. Lay representatives may have limited knowledge, and may transfer undesirable concerns to patients.^16^ Planning ahead and adjusting strategies to accommodate differing types of involvement desired by different groups of lay representatives are necessary for successful lay involvement.^17^, ^18^, ^19^

In the Norwegian context, group‐based education has traditionally been offered in hospitals, whereas the recent health political reforms promote self‐management‐supporting groups in local communities with lay‐led groups.^20^, ^21^ We have illustrated the Norwegian structure of group‐based support measures in Table 1.

**Table 1:** Norwegian structure of group‐based support measures

Public patient education programmes	Private, non‐profit, self‐management support groups	Local public physical activity and nutrition programmes
Professional‐led, developed and conducted in co‐operation with lay representatives	Lay‐driven groups conducted by patient organizations	Publicly initiated, both professional‐ and lay‐driven
Mostly performed in hospitals and policlinics, referral based	Performed in municipalities, diagnosis specific, membership based	Performed in municipalities, low‐threshold activities, not limited to diagnosis, open to all

Patients with type 2 diabetes are perceived to particularly benefit from group‐based self‐management support in Norway.^20^ In our study, we consider any group that offers health‐relevant activities (see Table 1) as group‐based self‐management support. Participants in our study are both individuals with type 2 diabetes who are or have been participating in group‐based activities and individ‐uals with no desire to join such groups.

Whilst a wide array of different group‐based activities and attendant benefits have been described in the literature,^22^, ^23^, ^24^, ^25^ reaching and engaging those likely to benefit from participation in group‐based activities for self‐management support remain insufficient.^26^, ^27^ There are recruitment challenges in engaging those patients who are most in need,^28^, ^29^ and non‐participation may contribute to inequality in the accessibility of support.^30^ However, patients' own perspectives related to the challenges associated with joining group‐based support have rarely been explored.^30^ In the current paper, we aim to investigate how individuals with type 2 diabetes understand how group‐based self‐management support may (or may not) help in accommodating the challenges of living with a long‐term condition. Why do some join while others refuse to participate in group‐based self‐management support?

## Theoretical approach

To approach how individuals with a chronic condition such as type 2 diabetes perceive group‐based self‐management support, we applied an institutional logic framework. We see the concept of institutional logic here as referring to underlying understandings of health and self‐management support, influenced by the wider societal policies and structures. Individuals produce and reproduce institutional logic in patterns of practices, assumptions and values through the process of negotiation, exchange and communication in group settings.^31^ Group participants are likely to draw on the institutional logic accessible to them in the wider society; in this case, understandings associated with perceptions of type 2 diabetes. In negotiations, individuals draw on dominant logic by focusing on certain themes of discussion rather than others, thereby producing the group dynamics that form the group identity. Through this theoretical lens, our research is within the epistemological stand of constructivism. The perspective of institutional logic thus has the potential to identify the logic related to participation or non‐participation in group‐based self‐management support.

## Design and methods

The study is anchored in a wider European collaboration project exploring the significance of social networks for self‐management support of chronic conditions in Europe.^32^ Through mutually decisions within the international project, the design of our study is fixed to a focus group‐based approach. As homogenous groups may lead to conformity and inhibit discussions,^33^ we aimed to recruit individuals who both attended and did not attend group‐based activities, to achieve a participant composition able to trigger views and contributions from all participants. Disagreement and co‐operation lead to a negotiated order as a product of social interaction.^34^ The focus group composition thus sets the stage for knowledge construction.^35^

We considered six participants in each focus group an ideal number that would allow everyone to contribute and be large enough to include varying opinions.^35^, ^36^ Our study presents data from three focus group interviews comprising a total of 16 individuals with type 2 diabetes in Norway.

### Recruitment

During the recruitment process, we established contact with groups initiated to support people with long‐term conditions in Norway (Table 1). Group education programmes and motivational groups led by the Norwegian Diabetes Association were contacted. A Healthy Life Centre located in an urban deprived part of Oslo referred patients who participated in some of their low‐threshold activities. In accordance with existing literature,^26^, ^27^ we experienced difficulties in reaching patients who did not want to join any groups. Specialist diabetes nurses in polyclinics were helpful in contacting these patients. It was, however, a time‐consuming activity, stretching the recruitment period from March 2013 to September 2013. As we did not wish to wait too long after receiving a suitable number of participants before conducting the actual interviews, we sat up the interview date as soon as we had six to eight participants available. Some of the participants did not attend to the focus group meeting. As a result of last minute redraw; two of the groups had four participants, while the third had eight. A total of 16 respondents participated, of which half were attending various group‐based activities and the other half did not (Table 2). Guidance on group size in focus group research goes seldom beyond a minimum of four participants^37^; there is even an indication that more information is obtained by conducting two groups of four participants than one group of eight.^38^ The value of having recruited the voices of patients characterized as difficult to reach in this field urged us to make the best of our focus groups.

**Table 2:** Focus group characteristics

	Group 1	Group 2	Group 3
Gender	1 woman 3 men	3 women 1 man	4 women 4 men
Mean age	55	58	68
Mean time since onset of diagnosis	6 years	8 years	14.5 years
Group affiliation	One individual joins several groups. The rest of the group participants have no group affiliations	Two individuals have group affiliations, and two have no group affiliations	Five individuals have group affiliations. Three individuals have no group affiliations
Non‐attendance	2	2	0
Location	Hospital patient education facility	Urban healthy life centre	Rural local centre where elderly meet

We formulated a written consent form and an invitation asking participants to reflect upon the benefits or concerns associated with group‐based activities for self‐management support, which all participants signed.

### The interviews

The group interviews were led by two moderators and were conducted in Norwegian. Notes on the interactions were taken both during and after the focus group interviews. The interview guide was semi‐structured with overarching main themes related to the value and role of group‐based activities as perceived by the participants and why people do or do not want to join groups.

At the beginning of each interview, the participants were encouraged to express both concordance and disagreement with others' statements and to communicate directly with each other.

### Data analysis

The group interviews were tape‐recorded and transcribed verbatim by the first author. We also translated the statements and discussions into English.

Following each interview, the researchers discussed their reflections. The authors read through the entire transcripts individually and looked for themes of agreement and conflict in each interview. The analysis was undertaken through discussions between the first author and the co‐authors (senior researchers). The on‐going discussion aimed at achieving agreement related to the themes of negotiation between group participants in addition to the groups' relational characteristics. Our understanding of a ‘natural’ data occurrence in the discussions is in accordance with a situated constructionist approach.^35^ The recurring themes of discussion are hence important as analytical points of departure. The opinions stated are not treated as belonging to the participants or as opinions held by the whole group but rather as understandings emerging and negotiated in the group context, influenced by the wider institutional logic available to the participants.^34^

We aimed to present our findings in a way that conveys a sense of the negotiation of meanings between the participants.^35^ The most engaging moments of discussions are presented alongside the recurring themes. We also present the focus groups' conversational and relational contexts to show how statements may be understood as being influenced by the group context.

## Findings

The groups were characterized by a high level of engagement and interaction flow, making it possible to let the discussion follow its own logic while staying relevant to our questions. An overwhelming amount of the discussions between participants dealt with how society sees type 2 diabetes. The diagnosis and group‐based self‐management support were repeatedly associated with the experience of stigma of a ‘lifestyle‐related disease’. In the following, we first present the participants' perceptions of stigma as a theme of consensus in all of the focus groups. Preceding each example, we describe the conversational context of the group to establish the setting for participants' statements.

### Establishing stigma

#### Group 1

The conversational context is characterized by participant K and T holding a dominant opinion against that of the group as a whole. The dominant opinion is continuously challenged by participant A, whereas participant N stands in between the dominant voices.

The researcher asks how others react when the patients reveal their diagnoses.

Man T Lazy slacker.

All of the participants are speaking simultaneously. The word ‘couch potato’ is mentioned, and woman A laughs out loud.

Man K Yes, rather that than a fat pig that sits on the couch the whole day.

Man T And someone who does not bother to exercise and just eats unhealthy, like ‘cheating food’.

All of the group participants are laughing out loud.

Man T It is obviously a negative association.

In the excerpt above, Man T starts out and triggers the others to follow up in an interaction flow of recognition that results in participants laughing out loud. The laughter may be understood as a way to lighten the tension related to the dominant voices speaking against each other in the group setting. Laugher is, however, also shown to function in focus groups as a tool for neutralizing challenging statements with an edge of humour.^39^ It is thus likely that the negative descriptions may have triggered a need to balance out feelings of shame, generating laughter, and could also be seen as expressing a supporting community (‘we’ and ‘the others’). As such, we can see the focus group expressing group‐based support between individuals. The engagement is visible through all of the participants taking part in the laughter sharing and producing confirming statements regarding the negative things being said about type 2 diabetes.

#### Group 2

Group 2 is characterized by participant E taking most of the space in the group discussion by steering the conversation towards his life‐experiences, which go beyond the content of the interview. The rest of the participants try to discuss the themes of the interview when given the turn and space to participate.

The researcher asks how people view chronic illness today.

Woman H Chronic illnesses are viewed as self‐inflicted chronic illnesses! I certainly believe that there is more of that now, like diabetes and COPD, and yes. (They are) self‐inflicted. That is what I have noticed.

The researcher asks if anybody else has noticed this.

Man E (First talks about his childhood experiences when his father had tuberculosis, and the children were not allowed to tell anyone, before he returns to the type 2 diabetes diagnosis.) It is a looser thing in a way. It's a stigma.

In the above excerpt, the group participants compare the stigma of having type 2 diabetes with taboos associated with illnesses connoted with shame.

#### Group 3

The conversational context is characterized by many of the participants having group affiliations and knowing each other. Participant A1, A2 and P do not have any group affiliations. The tone is easy going. The common ground relates to discussion of the GPs competence.

The researcher asks how the presentation of type 2 diabetes in the media affects the participants.

Woman K Diabetes type 2 is far more complicated than how it is being talked about by the public.

Woman A It is almost always presented as a life‐style disease, no matter if you got it through your genes or if you…

All of the participants start talking simultaneously, agreeing with woman A.

Woman K It is viewed as a life‐style disease. (…) It makes me angry.

Woman A The first thing my mother said when I got it was, ‘Really? But you are so slim!’

All of the participants make sounds of consent, and man H laughs.

All of the excerpts above present a sense of consensus, referring to prejudices associated with the diagnosis. All participants agree that type 2 diabetes is understood as a self‐inflicted disease. The examples show how the groups establish themselves as groups of ‘insiders’, meaning ‘we who know how it is to have type 2 diabetes’. The atmosphere is characterized by an awareness of how the world outside the group (‘outsiders’) looks at individuals with type 2 diabetes. Establishing stigma thus creates a collective ‘we’ in the focus groups. The disease is connoted with shame (Group 2), the public does not understand the complexity of the disease (Group 3), and others associate people with type 2 diabetes as being overweight (Group 3) and lazy (Group 1).

### Negotiating identity

An important strand of consensus in the focus groups is related to the construction of an identity as worthy individuals despite the stigma associated with type 2 diabetes. To participate in the construction of dignity, a negotiation of identity must occur. The construction of a collective group identity as worthy and responsible individuals opposes the implication of blame related to the logic of moral responsibility. The institutional logic of moral responsibility conveys the message that you are a morally weak individual if you do not comply with the rules of healthy eating and exercising.^40^ We find the logic of moral responsibility to be present and available for participants in our focus groups. We shall return to the negotiation of responsibility shortly.

In the following excerpt, we present participant discussions related to how participating in a group‐based support activity may strengthen an unwanted identity as ‘a patient with diabetes’. The opposite stand in the discussion highlights that having a group affiliation implies finding other ‘insiders’ who understand the complexity of having a chronic disease. Both stands show that having a group affiliation and not having one are mechanisms through which participants construct dignity.

#### Group 1

The researcher asks if it is good to join a group where the stigma is less obvious (the participants have just discussed that the disease is associated with laziness).

Man K Yes well… (Seems to disagree) But you know – then it is like – here I am, having a good time with my gang, we have all the same problems that I have, and we make it cosy. I think we have to be braver and dare to say to other people that having type 2 diabetes is not about all of the bad things people are saying.

All of the participants start talking simultaneously, and Man K continues without listening to them.

Man K Ninety percent of people who have type 2 diabetes are extremely focused on what kind of problems they have, and they really want to do something about it! Nobody else is as aware of these problems as we who actually have them.

Woman A But don't you think that you are just afraid? Joining a meeting within the Diabetes Association, for instance, is something totally different than you think.

Man T groans with dissatisfaction.

Man K It may be good to join other groups also, outside of the type 2 diabetes association…

Woman A But it is not…

Man K If you are happy with sitting in your garden and reading a book, then…

Man T Some like flowers, right? It is all individualized, what we like and don't like.

The discussion between Man K, Man T and Woman A continues. A while later, the fourth participant joins the discussion:

Man N I think it is good to get information and things like that. But to make a group with only patients, why should we do that? We are just normal people and want different things. Why should we have our own group? Some people have a beard; should we then have a group for people with beards?

All of the participants are laughing.

The excerpt above illustrates participants negotiating identity either as members of group‐based activities or as independent individuals who manage their health on their own. Not needing the group‐based support may be interpreted as a way to accommodate the need to not identify with the group and instead ‘dare to say to other people that having type 2 diabetes is not about all of the bad things that people are saying’, as Man K puts it. The dominant voice here is associated with being independent and coping with the disease on your own instead of ‘hiding’ in a group with other type 2 diabetes patients. Another way of interpreting this is not related to being strong but rather seeing the statements as a way of not letting the diagnosis identify you. Another statement from the same group shows the opposite perspective:

Woman A Earlier I was like that; for instance, when I had arthritis, I would always tell people that I had tendonitis or that I had sprained my foot and stuff like that, because I was not ready to actually admit to myself that I had the disease. (…) I would just push it away. And when people tried to help me, I would say to them ‘no, I can manage’, because I wanted to do everything on my own, I had to prove it for myself. So you kind of, you have to be motivated to actually accept help. It's all about people asking you if you want to join them, right. And when I actually started (joining a group), now I understand that it's good for me. And when I think back on all those years, where would I have been today if I just kept sitting inside… I would get depressed. You get all of these new impulses when you talk to other people.

The notion of ‘doing things on your own’ is important in both examples above as statements relating to being independent and responsible. Although the differing positions regarding joining groups or not are contrasted, both perspectives promote an image of patients as responsible, independent and worthy. When dealing with the stigma of a self‐inflicted disease, participants also negotiate responsibility. The alleged belief that individuals with type 2 diabetes are overweight, lazy and unintelligent is established, and then, as a response, the participants negotiate the sense of being responsible.

### Negotiating responsibility

The negotiation of responsibility is a matter that participants in the focus groups express ambivalence about. The differing positions in negotiating responsibility relate to whether the patients’ disease is a result of bad management habits, a lack of qualified competence in wider society, or genetic disposition. The contradiction is that while the participants are discussing a lack of competence in the society and among health professionals, they simultaneously maintain the opinion that the management of type 2 diabetes is solely their own responsibility. The notion of moral responsibility represents the driving force underlying negotiating responsibility.

While criticizing the wider society, the GP is presented as the main information provider who has the ability to enable patients to achieve proper illness management. If the GP fails in providing the necessary information, the challenges of exercising suitable self‐management become understandable. All of the group participants frequently engaged in discussions about the GP's roles and competencies:

#### Group 3

Woman (K) suspects that she has had the diagnosis for a much longer time than her doctor says:

Woman K Well, no one has found out anything, really. I am at the moment taking medication, but I must admit that I am actually considering something more (insulin). I miss more scientific competence in doctors. It's all just a big mess.

Another woman is whispering, barely noticeable: *I couldn't agree more*.

Woman K continues They know too little; if you ask them for something, they just look it up in a book (bangs her hand on the table as if she is looking something up in a book). Another participant in the group makes a sound of consent.

Man H (this participant has previously revealed that both his GP and his dentist have type 2 diabetes) replies I am lucky.

Woman K Yes, that is exactly why I said that, you are lucky! I really wish I could talk to my GP about my disease.

Woman L When I arrived at my GP's office with a specimen, he asked me what he was supposed to do with it!

Man H (laughs out loud) Really?

Several participants start talking simultaneously, supporting the statement about bad GPs.

The above excerpt shows that assigning low quality of care to insufficient diabetes competencies of the GP which is in accordance with earlier findings.^41^ GPs own experiences with illness in the example above are highly valued as providing additional competence to the medical approach, as also shown in earlier research.^12^ All of the participants in this focus group agree that Man H is lucky because he has a GP with type 2 diabetes. All of the other discussions regarding GPs describe negative experiences:

#### Group 1

Man T is emphasizing how the severity of type 2 diabetes is not easy for patients to understand: *My impression is that the GP I was going to at that time (onset of diagnosis), he didn't make me aware of all the things I should have been careful with. After that, it took med a long time to realize that the disease actually was dangerous!*

Man K Exactly, that it is dangerous! (Consent)

Man T It was only after I switched doctors that the second GP said to me that he will probably see me again in his office in some years, with a heart condition or myocardial infarction. So when you get type 2 diabetes – you almost think it is just an ordinary disease, and you don't understand the severity…

The researcher asks whether GPs should inform patients about the severity of type 2 diabetes.

Man T This information should come from a GP, yes.

Researcher But could it come from other people who also have the same diagnosis?

Woman A Yes.

Man T Yes, but then you wouldn't take it seriously.

With the following excerpts, we illustrate the ambivalence of participants referring to how being independent of the GP's competence is a way to present oneself as a responsible information seeker. All participants maintained a strong emphasis on their own responsibility, despite their GPs lacking the necessary information:

#### Group 1

Man T You have to make some kind of effort yourself…

Woman A That is exactly what I am saying; you have to do more than half of the job yourself.

Man T Yes.

Woman A We cannot expect…

Man T No, we cannot expect.

Woman A Just think about it. We are grown‐ups. We can't expect that someone will come and help us. (…) You have to be interested in doing something yourself.

#### Group 3

Man H (pointing his finger towards his own chest) I have the sole responsibility for my diabetes.

All of the participants make sounds of consent.

Man H If I need help from the doctor, then I have to call and ask for it; I cannot expect that someone will do it for me.

Participants highlight the GP's lack of competence regarding type 2 diabetes at the same time that they underline their own responsibility for managing their illness. The ambivalence is seen as reasonable when understood together with the strategy of both joining a group‐based activity and distancing oneself from group affiliations to present themselves as responsible self‐managers. We see the different strategies of allocating responsibility and joining groups or not joining groups as ways to create an illness dignity in order to appear as responsible individuals rather than as the ‘negligent diabetic’. The construction of a worthy identity is the result of a collective identity negotiation in the focus groups.

## Discussion

Our findings show that both joining a group‐based activity and distancing oneself from groups are strategies for handling the stigma of an allegedly self‐inflicted disease.

Earlier literature has focused on identity work regarding challenges related to the self‐management of type 2 diabetes. The differences between health professionals’ ‘disease orientation’ and patients’ ‘life over disease’ approach have been used to explain poor self‐management among type 2 diabetes patients.^4^, ^42^ Joining a group‐based activity for self‐management support may, for some, involve making the disease an important part of their identity. Group affiliation may therefore sound threatening to individuals who do not want to identify with having the disease because they prefer to identify as being independent and managing their health on their own. Another way to understand the withdrawal from groups may also be that group‐based activity is characterized by social comparisons, which do not fit well with patients who struggle the most.^43^ Nevertheless, our study shows that, for some, participating in group‐based support may strengthen their illness dignity.

Through the theoretical lens of institutional logic, we have found the discussions in the focus groups to be embedded with social, cultural and political structures, here represented by the growing focus on individual responsibility for health, guiding the identities and goals of the groups. Our study illustrates that contemporary self‐management policy has contributed to the institutional logic of (individual) moral responsibility that is accessible to participants in focus groups. The logic of moral responsibility for disease fits well in a health‐related political landscape characterized by individual responsibilities for health.^44^ Contemporary health promotion policies are described to reflect and reinforce a prevailing ideology of neoliberalism, operating towards the creation of a ‘good’ and ‘healthy’ citizen and making a modern health conscious movement.^45^ ‘As the burden of health care is reduced from the shoulders of the state, it is then placed upon the consciousness of individual citizens’.^45^ ^: 100^ In our study, the conscious awareness of individual responsibility for disease makes group affiliation intimidating rather than supportive for some participants. Faced with the presumed societal opinion of them, the participants negotiate a worthy identity in the focus groups, which we believe is likely to happen in group‐based activities where having an allegedly lifestyle‐related disease is a common circumstance.

Based on our results, the logic of moral responsibility seems to motivate both the participation in group‐based activities and the non‐participation. Crossley^46^ illustrated that resistance to health promotion is a result of health being interlayered with morality. She also found that resistance may reinforce inconvenient management habits. Interestingly, the primary need in our focus groups was dealing with notions of blame and responsibility. Gallant suggests that actively managing social influences is an important aspect of successful self‐management. In some instances, the causal influence between social support and self‐management may thus have a negative association.^47^

Considering the skewed recruitment to group‐based measures, our findings underline an inevitable inequality in access to support as a consequence of individualization and the ideals of free choice. Additionally, because several participants highlighted a lack of societal competence while simultaneously resisting group affiliation, there seems to be a pressing need for accessible sources of competent self‐management support. Norway has adapted systems of self‐management support based on strengthening individual motivation, knowledge, goal setting and problem‐solving, which are all individualized measures, conceptualizing self‐management as an individual capacity. To some, this conceptualization impedes the use of existing group‐based self‐management support. Our findings indicate a need to develop alternative measures to meet the needs of all patients with chronic conditions.

## Limitations and strengths

It is important to note that our findings include only discussions and statements expressed in focus groups. Focus groups are associated with dynamics directed both by the researcher and the questions asked. The situated negotiation being the object of our investigation, makes individual member checking as recommended in validity procedures within qualitative research^48^ problematic. Furthermore, the size and distribution of participants attending and not attending group‐based activities in each focus group may not have been optimal. Nevertheless, the strength of our research is the variety of included participants as we managed to recruit in terms of both individuals joining and not joining different group‐based activities. Because most of the groups were characterized by conversational flow, we believe that we succeeded in triggering discussions and engagement between participants. Furthermore, our findings are consistent with other studies within this field of research.^12^, ^41^, ^42^

As group‐based activities for self‐management support are increasingly important in European societies, our study contributes to the understanding of self‐management policies and their implications regarding the needs of patients with chronic conditions today. The knowledge of modern health policies triggering the need to counteract blame and construct an illness dignity is relevant to a wide audience and poses potential new research questions that may better address meeting the needs of people with chronic conditions.

## Conflicts of interest

The authors have no conflict of interests to declare.

## Funding

First author was funded by the Norwegian National Advisory Unit on Learning and Mastery in Health. The research was funded by the Seventh Framework Programme of the European Commission (Grant agreement no. 279081 ‘EU WISE’).

## References

[1] Epping‐Jordan JE , Pruitt SD , Bengoa R , Wagner EH . Improving the quality of health care for chronic conditions. Quality and Safety in Health Care, 2004; 13: 299–305. 15289634 10.1136/qshc.2004.010744 PMC1743863

[2] Kennedy A , Rogers A , Bower P . Support for self care for patients with chronic disease. British Medical Journal, 2007; 335: 968–970. 17991978 10.1136/bmj.39372.540903.94 PMC2071971

[3] Wagner EH , Groves T . Care for chronic diseases. British Medical Journal, 2002; 325: 913–914. 12399321 10.1136/bmj.325.7370.913 PMC1124427

[4] Foss C , Knutsen I , Kennedy A *et al* Connectivity, contest and the ties of self‐management support for type 2 diabetes: a meta‐synthesis of qualitative literature. Health & Social Care in the Community, 2015. doi:10.1111/hsc.12272. 10.1111/hsc.12272 26429546

[5] Michailakis D , Schirmer W . Agents of their health? How the Swedish welfare state introduces expectations of individual responsibility. Sociology of Health & Illness, 2010; 32: 930–947. 20649889 10.1111/j.1467-9566.2010.01262.x

[6] Ong BN , Rogers A , Kennedy A *et al* Behaviour change and social blinkers? The role of sociology in trials of self‐management behaviour in chronic conditions. Sociology of Health & Illness, 2014; 36: 226–238. 24528304 10.1111/1467-9566.12113

[7] Karlsen B , Oftedal B , Bru E . The relationship between clinical indicators, coping styles, perceived support and diabetes‐related distress among adults with type 2 diabetes. Journal of Advanced Nursing, 2012; 68: 391–401. 21707728 10.1111/j.1365-2648.2011.05751.x

[8] Hunt LM , Arar NH , Larme AC . Contrasting patient and practitioner perspectives in type 2 diabetes management. Western Journal of Nursing Research, 1998; 20: 656–676; discussion 77–82. 9842286 10.1177/019394599802000602

[9] Kennedy A , Rogers A , Vassilev I *et al* Dynamics and nature of support in the personal networks of people with type 2 diabetes living in Europe: qualitative analysis of network properties. Health Expectations, 2014; 18: 3172–3185. 25393694 10.1111/hex.12306 PMC5810651

[10] Bodenheimer T , Lorig K , Holman H , Grumbach K . Patient self‐management of chronic disease in primary care. Journal of the American Medical Association, 2002; 288: 2469–2475. 12435261 10.1001/jama.288.19.2469

[11] Taylor‐Robinson DC , Lloyd‐Williams F , Orton L , Moonan M , O'Flaherty M , Capewell S . Barriers to partnership working in public health: a qualitative study. PLoS ONE, 2012; 7: e29536. 22238619 10.1371/journal.pone.0029536 PMC3251584

[12] Whelan E . ‘No one agrees except for those of us who have it’: endometriosis patients as an epistemological community. Sociology of Health & Illness, 2007; 29: 957–982. 18092978 10.1111/j.1467-9566.2007.01024.x

[13] Griffiths C , Foster G , Ramsay J , Eldridge S , Taylor S . How effective are expert patient (lay led) education programmes for chronic disease? British Medical Journal, 2007; 334: 1254–1256. 17569933 10.1136/bmj.39227.698785.47 PMC1892511

[14] Goldman ML , Ghorob A , Eyre SL , Bodenheimer T . How do peer coaches improve diabetes care for low‐income patients? A qualitative analysis The Diabetes Educator, 2013; 39: 800–810. 24168838 10.1177/0145721713505779

[15] Joseph DH , Griffin M , Hall RF , Sullivan ED . Peer coaching: an intervention for individuals struggling with diabetes. The Diabetes Educator, 2001; 27: 703–710. 12212020 10.1177/014572170102700511

[16] Mandalia P , Stone M , Davies M , Khunti K , Carey M . Diabetes self‐management education: acceptability of using trained lay educators. Postgraduate Medical Journal, 2014; 90: 638–642. 25258417 10.1136/postgradmedj-2014-132865

[17] Armstrong N , Herbert G , Aveling EL , Dixon‐Woods M , Martin G . Optimizing patient involvement in quality improvement. Health Expectations, 2013; 16: e36–e47. 23374430 10.1111/hex.12039 PMC3883095

[18] Hogg C , Williamson C . Whose interests do lay people represent? Towards an understanding of the role of lay people as members of committees. Health Expectations, 2001; 4: 2–9. 11286594 10.1046/j.1369-6513.2001.00106.x PMC5060049

[19] Litva A , Canvin K , Shepherd M , Jacoby A , Gabbay M . Lay perceptions of the desired role and type of user involvement in clinical governance. Health Expectations, 2009; 12: 81–91. 19250154 10.1111/j.1369-7625.2008.00530.x PMC5060476

[20] Ministry of Health and Care Services . The Primary Health and Care Services of Tomorrow – Localised and Integrated. Ministry of Health and Care Services, 2015; 23: 2012–2013.

[21] Steinsbekk A , Rygg LO , Lisulo M , Rise MB , Fretheim A . Group based diabetes self‐management education compared to routine treatment for people with type 2 diabetes mellitus. A systematic review with meta‐analysis. BMC Health Services Research, 2012; 12: 213. 22824531 10.1186/1472-6963-12-213 PMC3418213

[22] Barlow J , Edwards R , Turner A . The experience of attending a lay‐led, chronic disease self‐management programme from the perspective of participants with multiple sclerosis. Psychology and Health, 2009; 24: 1167–1180. 20204986 10.1080/08870440802040277

[23] Barlow J , Wright C , Sheasby J , Turner A , Hainsworth J . Self‐management approaches for people with chronic conditions: a review. Patient Education and Counseling, 2002; 48: 177–187. 12401421 10.1016/s0738-3991(02)00032-0

[24] Davis KL , O'Toole ML , Brownson CA , Llanos P , Fisher EB . Teaching how, not what the contributions of community health workers to diabetes self‐management. The Diabetes Educator, 2007; 33: 208S–215S. 17620403 10.1177/0145721707304133

[25] Nation H . How effective are expert patient (lay led) education programmes for chronic disease? British Medical Journal, 2007; 334: 1255. 10.1136/bmj.39227.698785.47 PMC1892511 17569933

[26] Cauch‐Dudek K , Victor JC , Sigmond M , Shah BR . Disparities in attendance at diabetes self‐management education programs after diagnosis in Ontario, Canada: a cohort study. BMC Public Health, 2013; 13: 85. 23360373 10.1186/1471-2458-13-85 PMC3568403

[27] Mielck A , Reitmeir P , Rathmann W . Knowledge about diabetes and participation in diabetes training courses: the need for improving health care for diabetes patients with low SES. Experimental and Clinical Endocrinology & Diabetes, 2006; 114: 240–248. 16804798 10.1055/s-2006-924228

[28] Bury M , Pink D . The HSJ debate. Self‐management of chronic disease doesn't work. Health Service Journal, 2005; 115: 18–19. 15799275

[29] Foster G , Taylor SJ , Eldridge SE , Ramsay J , Griffiths CJ . Self‐management education programmes by lay leaders for people with chronic conditions. Cochrane Database of Systematic Reviews, 2007; 4: CD005108. 10.1002/14651858.CD005108.pub2 17943839

[30] Sandaunet AG . The challenge of fitting in: non‐participation and withdrawal from an online self‐help group for breast cancer patients. Sociology of Health & Illness, 2008; 30: 131–144. 18254837 10.1111/j.1467-9566.2007.01041.x

[31] Thornton PH , Ocasio W , Lounsbury M . The Institutional Logics Perspective: A new Approach to Culture, Structure, and Process. Oxford: Oxford University Press, 2012.

[32] Rogers A , Vassilev I , Sanders C *et al* Social networks, work and network‐based resources for the management of long‐term conditions: a framework and study protocol for developing self‐care support. Implementation Science, 2011; 6: 56. 21619695 10.1186/1748-5908-6-56 PMC3120720

[33] Kitzinger J . Focus groups In: Holloway I, (ed.) Qualitative Research in Health Care. 3rd edn Maidenhead: McGraw‐Hill International (UK) Ltd., 2007.

[34] Lehoux P , Poland B , Daudelin G . Focus group research and “the patient's view”. Social Science & Medicine, 2006; 63: 2091–2104. 16797811 10.1016/j.socscimed.2006.05.016

[35] Freeman T . ‘Best practice'in focus group research: making sense of different views. Journal of Advanced Nursing, 2006; 56: 491–497. 17078825 10.1111/j.1365-2648.2006.04043.x

[36] Kitzinger J . The methodology of Focus Groups: the importance of interaction between research participants. Sociology of Health & Illness, 1994; 16: 103–121.

[37] Carlsen B , Glenton C . What about N? A methodological study of sample‐size reporting in focus group studies. BMC Medical Research Methodology, 2011; 11: 26. 21396104 10.1186/1471-2288-11-26 PMC3061958

[38] Fern EF . The use of focus groups for idea generation: the effects of group size, acquaintanceship, and moderator on response quantity and quality. Journal of Marketing Research, 1982; 19: 1–13.

[39] Mkandawire‐Valhmu L , Stevens PE . The critical value of focus group discussions in research with women living with HIV in Malawi. Qualitative Health Research, 2010; 20: 684–696. 19926798 10.1177/1049732309354283

[40] Bossy D , Knutsen IR , Rogers A , Foss C . Institutional logic in self‐management support: coexistence and diversity. Health & Social Care in the Community, 2015; doi: 10.1111/hsc.12277. 10.1111/hsc.12277 26429669

[41] Lauvergeon S , Mettler D , Burnand B , Peytremann‐Bridevaux I . Convergences and divergences of diabetic patients' and healthcare professionals' opinions of care: a qualitative study. Health Expectations, 2015; 18: 111–123. 23121596 10.1111/hex.12013 PMC5060754

[42] Zoffmann V , Kirkevold M . Life versus disease in difficult diabetes care: conflicting perspectives disempower patients and professionals in problem solving. Qualitative Health Research, 2005; 15: 750–765. 15961873 10.1177/1049732304273888

[43] Rogers A , Gately C , Kennedy A , Sanders C . Are some more equal than others? Social comparison in self‐management skills training for long‐term conditions. Chronic Illness, 2009; 5: 305–317. 19933248 10.1177/1742395309350384

[44] McGregor S . Neoliberalism and health care. International Journal of Consumer Studies, 2001; 25: 82–89.

[45] Ayo N . Understanding health promotion in a neoliberal climate and the making of health conscious citizens. Critical Public Health, 2012; 22: 99–105.

[46] Crossley ML . ‘Could you please pass one of those health leaflets along?': exploring health, morality and resistance through focus groups. Social Science & Medicine, 2002; 55: 1471–1483. 12231023 10.1016/s0277-9536(01)00265-9

[47] Gallant MP . The influence of social support on chronic illness self‐management: a review and directions for research. Health Education & Behavior, 2003; 30: 170–195. 12693522 10.1177/1090198102251030

[48] Creswell JW , Miller DL . Determining validity in qualitative inquiry. Theory into Practice, 2000; 39: 124–130.

